# Effects of Tangshen Formula on urinary and plasma liver-type fatty acid binding protein levels in patients with type 2 diabetic kidney disease: post-hoc findings from a multi-center, randomized, double-blind, placebo-controlled trial investigating the efficacy and safety of Tangshen Formula in patients with type 2 diabetic kidney disease

**DOI:** 10.1186/s12906-016-1228-4

**Published:** 2016-07-26

**Authors:** Xin Yang, Bingxuan Zhang, Xiaoguang Lu, Meihua Yan, Yumin Wen, Tingting Zhao, Ping Li

**Affiliations:** 1 Beijing Key Lab for Immune-Mediated Inflammatory Diseases, Institute of Clinical Medical Science, China-Japan Friendship Hospital, 2 East Yinghua Road, Chaoyang District, Beijing 100029 China; 2 Beijing University of Chinese Medicine, 11 North Third Ring Road East, Beijing, 100029 China; 3 Department of Nephrology, Guang’anmen Hospital, China Academy of Chinese Medical Sciences, 5 Bei Xiange, Xicheng District, Beijing 100053 China

**Keywords:** Chinese herbal medicine, Tangshen formula, Liver-fatty acid binding protein, Diabetic kidney disease

## Abstract

**Background:**

Tangshen Formula (TSF) is a traditional Chinese medicine for the treatment of diabetic kidney disease (DKD). Liver-type fatty acid binding protein (L-FABP) is expressed in various tissues, including the kidney, where it is known as urinary L-FABP. Other studies demonstrated that urinary L-FABP may be a useful biomarker for monitoring DKD. This post-hoc analysis and cross-sectional study evaluated the changes in urinary L-FABP in DKD patients treated with TSF and conventional medicine.

**Methods:**

Post-hoc analysis was conducted on a multicenter, randomized, double-blind, placebo-controlled trial. A total of 180 participants with DKD including 98 with microalbuminuria and 82 with macroalbuminuria were enrolled in the original study. In addition to conventional treatment, 122 participants were randomly assigned to receive TSF and 58 to receive placebo. After 24-weeks of treatment, the intention-to-treat population in microalbuminuria stage was 56 in the TSF group and 25 in the placebo group, and in the macroalbuminuria stage 42 and 19, respectively. The primary outcome in the original trial was urinary protein level. In the current study, urinary and plasma L-FABP levels were measured in 30 microalbuminuria patients (15 in the TSF group and 15 in the placebo group) and 30 macroalbuminuria patients (15 in the TSF group and 15 in the placebo group). In addition, another 30 patients with normoalbuminuria (urinary albumin excretion rate (UAER) < 20 μg/min) were recruited for the cross-sectional study.

**Results:**

(1) In microalbuminuria patients, UAER in the TSF group displayed a significant decrease after 24 weeks of treatment (*P* = 0.045). Levels of urinary L-FABP in the TSF group were markedly lower than in the placebo group after 12 and 24 weeks (*P* = 0.004 and *P* = 0.047, respectively). (2) In macroalbuminuria patients, 24-h urinary protein levels decreased significantly compared with baseline in the TSF group at week 12 (*P* = 0.042) and week 24 (*P* = 0.041). The TSF group showed a significant decrease in urinary L-FABP after 12 and 24 weeks (*P* = 0.036 and *P* = 0.046, respectively). (3) Levels of urinary L-FABP increased markedly, correlating with severity of DKD. L-FABP in patients with normoalbuminuria, microalbuminuria, and macroalbuminuria were 5.9 (5.2, 7.8) μg/ml, 11.4 (6.8, 13.4) μg/ml and 18.5 (10.9, 23.4) μg/ml, respectively (*P* = 0.000).

**Conclusions:**

TSF combined with conventional therapy appeared to be effective in reducing urinary protein and urinary L-FABP. Urinary L-FABP levels appear to be associated with the severity of DKD.

**Trial registration:**

Chinese Clinical Trial Registry ChiCTR-TRC-10000843. Registered 15 April 2010.

## Background

Diabetic kidney disease (DKD) is a critical problem, affecting approximately one third of people with diabetes mellitus (DM) [1]. As the number of persons with DM increases [2, 3], there has been a concomitant rise in DKD prevalence with associated cardiovascular mortality and end-stage renal disease (ESRD) [4]. DKD is also known as diabetic nephropathy. It is characterized by a pathologic series of structural abnormalities of all renal compartments, including glomerular and tubular basement membrane thickening, mesangial expansion, interstitial inflammation, glomerular and tubular hypertrophy, glomerulosclerosis, and tubulointerstitial fibrosis [5, 6], which eventually lead to renal dysfunction [7].

It is now widely accepted that the tubulointerstitium is closely involved in the pathogenesis of diabetic nephropathy and its degree of fibrosis corresponds with the rate of decline in kidney function [8, 9]. In the last decade, clinical studies have demonstrated that increased urinary liver-type fatty acid binding protein (L-FABP), which is expressed in the proximal tubules in the human kidney, is associated with the severity and clinical prognosis of DKD [10]. For example, Kamijo et al. [11] found that urinary L-FABP levels were progressively increased in patients with normo-, micro-, or macroalbuminuria and further increased in those with ESRD. After a 4-year follow-up, urinary L-FABP levels in patients who showed DKD progression were higher than those without progression. Thus, urinary L-FABP might be a suitable biomarker for predicting and monitoring deterioration of renal function in DKD. Furthermore, an accumulating number of interventional studies have reported that urinary L-FABP responds to renoprotective treatment [12–14].

Traditional Chinese Medicine (TCM) has been used for millennia in China to treat a variety of diseases. Records of using TCM to treat diabetes and its complications can be found in ancient TCM literature. Even in modern China, TCM is a primary or complementary therapy for kidney disease [15, 16]. The traditional Chinese herbal formula, Tangshen Formula (TSF), is composed of several herbs and formulated based on empirical evidence. In our previous studies, TSF showed a beneficial effect in attenuating development of DKD [17–21]. In light of those results, we conducted a multicenter randomized double-blind placebo-controlled trial to further demonstrate the efficacy and safety of TSF in treating DKD in persons with type 2 diabetes [22]. Since L-FABP is a novel biomarker for prognosis of DKD, we conducted this post-hoc analysis to evaluate the effect of TSF on urinary and plasma L-FABP in different DKD stages. In addition, we enrolled another 30 sex- and age-matched patients with normoalbuminuria to validate the clinical relevance of urinary L-FABP levels at various stages of DKD.

## Methods

### Study design

This post-hoc analysis was conducted using data from a previously published multicenter randomized double-blind placebo-controlled study that investigated the efficacy and safety of TSF in patients with type 2 diabetic kidney disease (Chinese Clinical Trial Registry ChiCTR-TRC-10000843) [22]. During that study, peripheral blood and urine samples of the participants were collected at baseline, 12 weeks, and 24 weeks and had been in frozen storage at −80 °C. We used these blood and urine samples to conduct this post-hoc analysis on L-FABP. Details of the the original trial design have been published previously [22]. The original trial was approved by the ethics committee of the China-Japan Friendship Hospital (No.2006–059). All participants in the trial signed written informed consent documents.

### Diagnostic standards

Type 2 diabetes was diagnosed based on the American Diabetes Association guidelines (ADA; 2006) [23]. Diabetic kidney disease was diagnosed according to criteria of the National Kidney Foundation Kidney Disease Outcomes Quality Initiative (NKF-KDOQI; 2007) [24]. Participants also underwent TCM diagnosis for their presenting syndrome (that is, TCM signs and symptoms). The predominant syndrome of *qi*-*yin* deficiency combined with blood stasis was based on criteria established in *Clinical Research of New Investigational Drugs in Traditional Chinese Medicine* [25].

### Study population

The inclusion and exclusion criteria have been described previously [22] and are summarized here. Men and women between the ages of 25 and 75 with type 2 diabetes were eligible if they had urinary albumin excretion rate (UAER) between 20 and 200 μg/min, and/or 24-h urinary protein (24 h UP) between 0.5 and 2.0 g/d, and estimated glomerular filtration rate (eGFR) between 60 ml/min and 130 ml/min, fasting blood glucose (FBG) ≤ 7.8 mmol/L, and hemoglobin A1C ≤ 7.5 %; blood pressure < 140/90 mmHg; and TCM syndrome was deficiency of both *qi* and *yin* with blood stasis.

Patients were excluded if they had history of either kidney disease or other disease with elevated urinary protein, endocrine or metabolic disease, liver disease, cardiovascular disease within the past 3 months, or triglyceride (TG) > 10 mmol/L (>886 mg/dl). Patients taking glucocorticosteroids, thiazide diuretics, or niacin within the past 3 months were also excluded as were pregnant or breastfeeding women.

### Randomization

A random allocation sequence was generated by computer based on blocks of 6. Patients were randomly assigned to either the TSF group (conventional treatments plus TSF) or the placebo group (conventional treatments plus placebo) in a 2:1 ratio and classified according to their different stages of DKD (microalbuminuria or macroalbuminuria). A total of 191 patients were screened, resulting in enrollment of 180 participants, including 98 with microalbuminuria and 82 with macroalbuminuria; 122 were randomly assigned to receive TSF and 58 to receive placebo. After 24-weeks’ clinical observation, the intention-to-treat population in microalbuminuria stage was comprised of 56 participants in the TSF group and 25 in the placebo group, while there were 42 and 19 participants in the TSF and placebo groups, respectively, in the macroalbuminuria stage. Blood and urine samples were collected at baseline, 12 and 24 weeks and stored at −80 °C for further analyses. Since only 15 macroalbuminuria participants in the placebo group permitted their blood and urine to be collected, 30 sex- and age-matched microalbuminuria participants (15 in the TSF group and 15 in the placebo group) and 30 sex- and age-matched macroalbuminuria participants (15 in the TSF group and 15 in the placebo group) were included to assay their urinary and plasma L-FABP levels. In addition, 30 sex- and age-matched participants patients with normoalbuminuria (UAER < 20 μg/min) were recruited from China to Japan Friendship Hospital for a cross-sectional study.

### Treatment protocol

The treatment protocol has been described previously [22]. Based on ADA 2006 recommendations [23], antihypertensive treatment, glycemic control, and antilipemic agents were adopted as conventional treatment using open-label drugs (calcium channel blockers, insulin, statins). All participants received conventional treatment and participants with albuminuria received either an angiotensin-converting enzyme inhibitor or angiotensin receptor blocker agent. After a 2-week run-in period with diet control and programmed daily exercise, TSF and placebo were initiated for both groups with 8 g of granules dissolved in warm water, taken twice daily for 24 weeks.

The Chinese herbal formula TSF consists of seven natural herbs: astragalus root (*Astragalus membranaceus* (Fisch.) Bunge); rehmannia root (*Rehmannia glutinosa* (Gaertn.) DC.); notoginseng root (*Panax notoginseng* (Burk.) F.H. Chen); winged burning bush twig (*Euonymus alatus* (Thunb.) Sieb*.*); cornus fruit (*Cornus officinalis* Sieb. et Zucc.); rhubarb root and rhizome (*Rheum palmatum* L.); and bitter orange (*Citrus aurantium* L*.*). Main ingredients of the placebo were lactose, maltodextrin, and artificial food coloring. Preparation of TSF and placebo has been described previously [22]. TSF and placebo granules were identical in packaging, appearance, shape, size, and color.

### Outcomes assessments

In the original trial, the primary outcome measure was urinary protein level, which was assessed by UAER for participants with microalbuminuria and 24 h UP for participants with macroalbuminuria. Secondary outcome measures were renal function, including eGFR, Scr, and BUN, and lipid profile, including TG, total cholesterol (TC), low density lipoprotein (LDL), high density lipoprotein (HDL). Safety indicators included alanine transaminase (ALT) and aspartate aminotransferase (AST). In addition, FBG and A1C were assessed. Blood and urine samples were collected from participants and frozen at −80 °C at baseline, 12 and 24 weeks. For this post-hoc analysis, both urinary and plasma L-FABP levels were measured using human L-FABP ELISA kits (RapidBio Lab, Calabasas, CA, USA).

### Statistical analysis

Statistical analysis was performed with SPSS 13.0 software (IBM, Armonk, NY, USA). Normally distributed variables were expressed as mean ± SD. Abnormally distributed variables were expressed as medians (interquartile range (IQR)). To compare 2 normally distributed variables in the same group, a paired *t* test was used. Student’s *t* test was used for analyzing the difference of variables between 2 groups with normal distribution. In the 3 DKD groups, normally distributed variables were compared using one-way ANOVA, and all comparisons between abnormally distributed parameters were performed with the Kruskal-Wallis test. In TSF treatment trial, normality was assessed using the Kolmogorov–Smirnov test, levels of abnormally distributed urinary and plasma L-FABP were exponentially- transformed (ln-transformed) before analysis, and if data were normally distributed after transformation, Student’s *t* test was used to compare differences between 2 groups. Differences with *P* < 0.05 were considered statistically significant.

## Results

### TSF treatment trial

#### Baseline characteristics

Baseline demographic, clinical, and laboratory characteristics of participants with microalbuminuria and macroalbuminuria in the TSF and placebo groups were similar (*P* > 0.05) (Table 1).

**Table 1:** Baseline characterstics of participating diabetic kidney disease patients

	Microalbuminuria			Macroalbuminuria		
	TSF (*n* = 15)	Placebo (*n* = 15)	*P* Value	TSF (*n* = 15)	Placebo (*n* = 15)	*P* Value
Age (yr)	56.1 ± 12.5	51.8 ± 8.5	.382	55.5 ± 8.2	58.0 ± 10.8	.522
Male/Female	9/6	7/8	.384	6/9	5/10	.400
BMI (kg/m^2^)	26.1 ± 3.7	26.0 ± 3.9	.950	25.9 ± 3.0	27.3 ± 2.9	.271
Blood pressure						
Systolic (mmHg)	131.9 ± 7.8	128.3 ± 5.6	.259	130.1 ± 8.3	131.1 ± 9.3	.777
Diastolic (mmHg)	81.6 ± 6.4	76.2 ± 10.1	.142	78.6 ± 7.4	82.0 ± 4.9	.231
Laboratory variables						
FBG (mmol/L)	6.7 ± 0.9	5.7 ± 1.0	.097	6.9 ± 1.5	6.8 ± 0.7	.752
A1C (%)	6.7 ± 0.6	6.5 ± 0.6	.065	6.7 ± 1.0	6.5 ± 1.3	.579
TG (mmol/L)	2.5 ± 1.6	2.2 ± 1.2	.617	2.7 ± 1.8	2.2 ± 1.0	.528
TC (mmol/L)	5.2 ± 0.9	4.9 ± 1.6	.532	5.2 ± 0.9	5.6 ± 1.2	.545
LDL (mmol/L)	3.0 ± 0.9	3.2 ± 1.4	.729	3.0 ± 1.2	3.8 ± 1.6	.293
HDL (mmol/L)	1.3 ± 0.6	1.0 ± 0.3	.091	1.3 ± 0.4	1.5 ± 0.3	.182
ALT (U/L)	26.2 ± 12.6	22.2 ± 9.1	.417	22.2 ± 8.7	22.9 ± 7.8	.835
AST (U/L)	22.4 ± 7.8	17.2 ± 5.5	.102	20.3 ± 5.7	22.5 ± 8.5	.443

Values expressed as mean ± SD

Abbreviations: *ALT* alanine aminotransferase, *A1C* glycosylated hemoglobin, *AST* aspartate aminotransferase, *BMI* body mass index, *FBG* fasting blood glucose, *HDL* high density lipoprotein, *LDL* low density lipoprotein, *TC* total cholesterol, *TG* triglyceride

#### Effects of TSF on renal function indices and urinary and plasma L-FABP levels in DKD patients with microalbuminuria

**Fig. 1:**
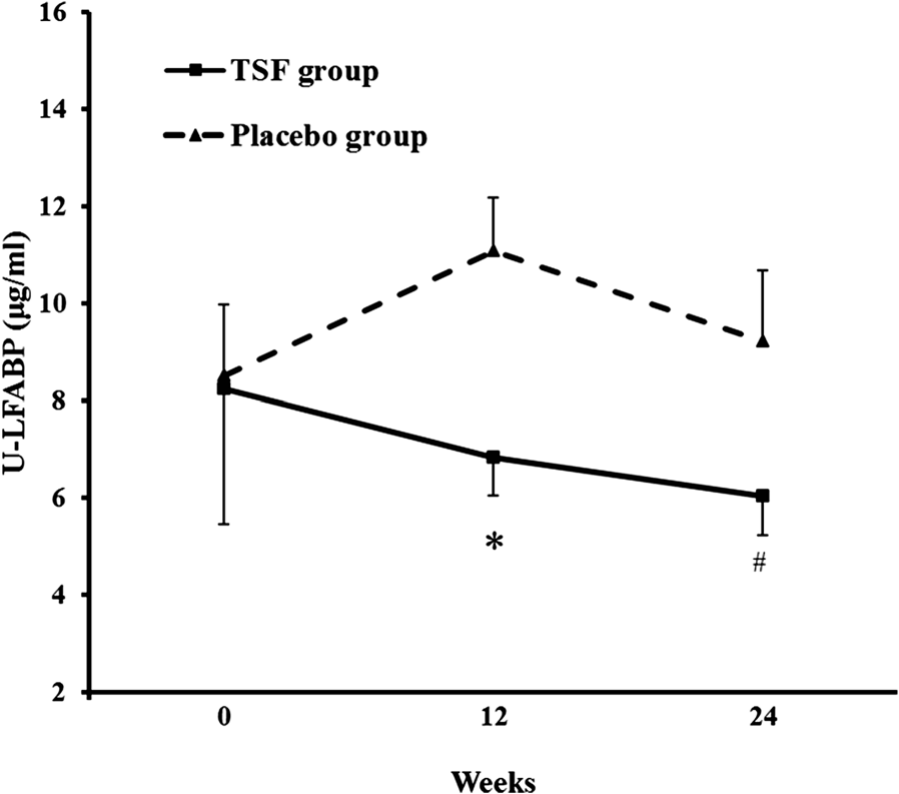
Effects of TSF on urinary L-FABP levels in microalbuminuria patients during the 24-week treatment period. Values presented as mean ± SE; *P = 0.004 and # *P* = 0.047 compared with the placebo group at the same time point. L-FABP = liver-type fatty acid binding protein

There was no statistical differences in levels of Scr, BUN, UAER, eGFR between the TSF group and the placebo group in participants with microalbuminuria at baseline (*P* = 0.136, *P* = 0.295, *P* = 0.975, *P* = 0.242, respectively), 12 weeks (*P* = 0.817, *P* = 0.080, *P* = 0.979, *P* = 0.713, respectively) and 24 weeks (*P* = 0.343, *P* = 0.484, *P* = 0.385, *P* = 0.697, respectively). However, after 12 and 24 weeks of TSF treatment, UAER decreased markedly when compared with that at baseline in the same group (*P* = 0.090 and *P* = 0.045), while in the placebo group, little or no change was exhibited (*P* = 0.199 and *P* = 0.259). Levels of Scr also declined and eGFR was increased over baseline in the TSF group although without significant difference (at 12 weeks, *P* = 0.222 and *P* = 0.354, respectively; at 24 weeks, *P* = 0.386 and *P* = 0.524, respectively). In the placebo group, Scr and eGFR were unchanged (at 12 weeks, *P* = 0.788, *P* = 0.860; at 24 weeks, *P* = 0.860, *P* = 0.713). Levels of plasma L-FABP were abnormally distributed in the TSF and placebo groups (*P* < 0.05), After ln-transformation, levels of ln plasma L-FABP in each group were normally distributed (*P* > 0.05). Compared with the placebo group, levels of ln plasma L-FABP in the TSF group showed no significant difference after 12 weeks or 24 weeks of treatment (*P* = 0.190 at baseline, *P* = 0.131 at 12 weeks, and *P* = 0.710 at 24 weeks, respectively) (Table 2). However, urinary L-FABP levels in the TSF group were significantly lower than levels in the placebo group after both12 weeks and 24 weeks of treatment (8.2 ± 10.1 μg/ml compared with 8.5 ± 4.4 μg/ml at baseline, *P* = 0.940, 6.8 ± 2.9 μg/ml compared with 11.1 ± 3.3 μg/ml at 12 weeks, *P* = 0.004 and 6.0 ± 3.0 μg/ml compared with 9.2 ± 4.9 μg/ml at 24 weeks, *P* = 0.047, respectively) (Fig. 1).

**Table 2:** Effect of TSF and placebo on Scr, BUN, UAER, eGFR and ln plasma L-FABP levels in patients with microalbuminuria

Parameters	Groups	Baseline	Week 12	Week 24
UAER (μg/min)	TSF	157.6 ± 72.9	115.9 ± 50.7	104.0 ± 32.4^a^
	Placebo	158.6 ± 99.0	116.5 ± 69.7	121.8 ± 69.7
BUN (mmol/L)	TSF	5.1 ± 1.2	5.6 ± 1.4	5.5 ± 1.4
	Placebo	5.7 ± 1.6	6.6 ± 1.5	6.0 ± 2.3
Scr (μmol/L)	TSF	73.5 ± 19.3	64.4 ± 19.7	67.6 ± 16.7
	Placebo	60.8 ± 18.2	62.7 ± 19.8	61.9 ± 15.1
eGFR (ml/min/1.73 m^2^)	TSF	102.7 ± 39.2	118.3 ± 49.4	113.9 ± 53.5
	Placebo	128.3 ± 61.0	124.8 ± 45.2	121.0 ± 44.0
ln plasma L-FABP (μg/ml)	TSF	1.0 ± 0.9	1.3 ± 0.8	1.2 ± 0.7
	Placebo	1.4 ± 0.7	1.7 ± 0.6	1.1 ± 0.7

All values expressed as mean ± SD

^a^: *P* < 0.05 compared with baseline of the same group (Paired *t* test)

Abbreviations: *BUN* blood urea nitrogen, *eGFR* estimated glomerular filtration rate, *ln plasma L-FABP* ln-transformed plasma liver-type fatty acid binding protein, *Scr* serum creatinine, *UAER* urinary albumin excretion rate

#### Effects of TSF on renal function indices and urinary and plasma L-FABP levels in DKD patients with macroalbuminuria

**Fig. 2:**
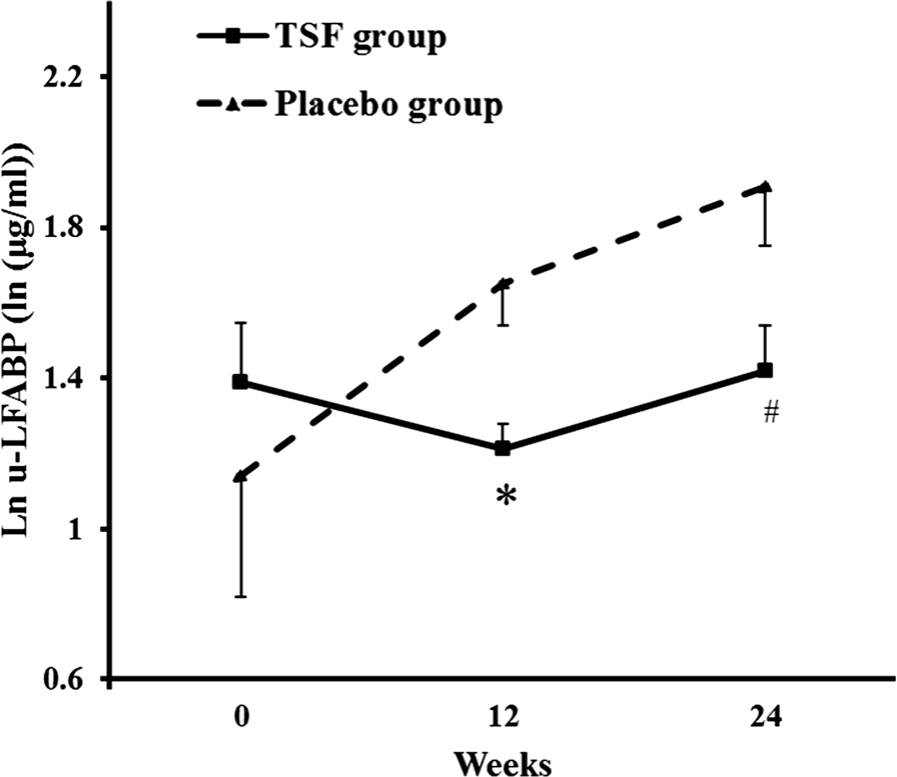
Effects of TSF on urinary L-FABP levels in macroalbuminuria patients during the 24-week treatment period. Values presented as mean ± SE; *P = .036 and # *P* = 0.046 compared with the placebo group at the same time point. ln urinary L-FABP = ln-transformed liver-type fatty acid binding protein

Levels of Scr, BUN, 24 h UP, eGFR were similar between the two groups in participants with macroalbuminuria at baseline (*P* = 0.594, *P* = 0.875, *P* = 0.996, *P* = 0.562, respectively), 12 weeks (*P* = 0.144, *P* = 0.158, *P* = 0.628, *P* = 0.376, respectively) and 24 weeks (*P* = 0.159, *P* = 0.090, *P* = 0.383, *P* = 0.646, respectively). In the TSF group, 24 h UP decreased significantly from 1.0 ± 0.7 g to 0.5 ± 0.5 g after 12 weeks (*P* = 0.042), remained at 0.5 ± 0.5 g after 24 weeks (*P* = 0.041). However, in the placebo group, little change was exhibited during the study period (*P* = 0.192 at 12 weeks and *P* = 0.415 at 24 weeks). In addition, Scr, BUN and eGFR remained unchanged compared to baseline in both the TSF group (*P* = 0.776, *P* = 0.979 and *P* = 0.911 at 12 weeks; *P* = 0.849, *P* = 0.998 and *P* = 0.538 at 24 weeks, respectively) and placebo group (*P* = 0.495, *P* = 0.119 and *P* = 0.664 at 12 weeks; *P* = 0.948, *P* = 0.113 and *P* = 0.914 at 24 weeks, respectively). Levels of plasma and urinary L-FABP were abnormally distributed in the TSF and placebo groups (*P* < 0.05). After ln-transformation, ln plasma and urinary L-FABP were normally distributed (*P* > 0.05). Ln plasma L-FABP showed little change in both the TSF and placebo groups (*P* = 0.643 at baseline, *P* = 0.328 at 12 weeks and *P* = 0.402 at 24 weeks, Table 3). However, ln urinary L-FABP decreased during 24 weeks of TSF treatment and significantly increased during 24 weeks of placebo treatment (baseline, TSF group 1.4 ± 0.6 μg/ml compared with placebo group 1.1 ± 1.0 μg/ml, *P =* 0.455; 12 weeks, TSF group 1.2 ± 0.3 μg/ml compared with placebo group 1.7 ± 0.3 μg/ml, *P* = 0.036; 24 weeks, TSF group 1.4 ± 0.5 μg/ml compared with placebo group 1.9 ± 0.5 μg/ml, *P* = 0.046) (Fig. 2).

**Table 3:** Effect of TSF and placebo on Scr, BUN, UAER, eGFR and ln plasma L-FABP levels in patients with macroalbuminuria

Parameters	Groups	Baseline	Week 12	Week 24
24 h UP (g/24 h)	TSF	1.0 ± 0.7	0.5 ± 0.5^a^	0.5 ± 0.5^a^
	Placebo	0.9 ± 0.6	0.6 ± 0.6	0.7 ± 0.7
BUN (mmol/L)	TSF	6.0 ± 1.7	6.0 ± 1.7	6.0 ± 1.9
	Placebo	6.1 ± 1.1	6.9 ± 1.6	7.2 ± 1.7
Scr (μmol/L)	TSF	74.9 ± 20.7	72.6 ± 22.6	73.5 ± 18.9
	Placebo	79.4 ± 18.2	84.0 ± 17.4	83.1 ± 16.4
eGFR (ml/min/1.73 m^2^)	TSF	104.1 ± 32.8	102.7 ± 37.7	97.3 ± 25.8
	Placebo	96.2 ± 30.0	91.3 ± 30.1	92.3 ± 32.2
ln plasma L-FABP (μg/ml)	TSF	1.7 ± 1.0	1.3 ± 1.3	1.5 ± 0.9
	Placebo	1.6 ± 1.2	0.8 ± 1.4	1.2 ± 1.0

All values expressed as mean ± SD

^a^ *P* < 0.05 compared with baseline of the same group (Paired *t* test)

Abbreviations: *BUN* blood urea nitrogen, *eGFR* estimated glomerular filtration rate, *ln-serum L-FABP* ln-transformed serum liver-type fatty acid binding protein, *Scr* serum creatinine, *UAER* urinary albumin excretion rate

### Cross-sectional study

#### Urinary L-FABP and Plasma L-FABP levels in DKD patients

Levels of urinary L-FABP in each DKD group differed significantly from levels in the other groups and increased markedly in accordance with the severity of DKD (*P* = 0.000 between three groups; DKD patients with microalbuminuria compared with DM patients with normoalbuminuria *P* = 0.002; DKD patients with macroalbuminuria compared with DM patients with normoalbuminuria *P* = 0.000; DKD patients with macroalbuminuria compared with DM patients with microalbuminuria *P* = 0.005, respectively) (Table 4). However, plasma L-FABP levels did not differ significantly between each DKD group (*P* = 0.888).

**Table 4:** Urinary and plasma L-FABP levels in patients in different stages of DKD

Groups	Urinary L-FABP (μg/ml)	Plasma L-FABP (μg/ml)
	Median (IQR)	Median (IQR)
DM patients with normoalbuminuria (*n* = 30)	5.9 (5.2, 7.8)	4.4 (2.9,11.3)
DKD patients with microalbuminuria (*n* = 30)	11.4 (6.7, 13.4)^a^	6.7 (3.4,11.4)
DKD patients with macroalbuminuria (*n* = 30)	18.8 (10.9, 23.4)^a,b^	8.6 (2.5,12.4)

^a^ *P <* 0.05 compared with the DM patients with normoalbuminuria (Kruskal-Wallis test)

^b^ *P* < 0.05 compared with the DKD patients with microalbuminuria (Kruskal-Wallis test)

Abbreviations: *IQR* interquartile range, *serum L-FABP* serum liver-type fatty acid binding protein, *urinary L-FABP* urinary liver-type fatty acid binding protein

## Discussion

The renoprotective effects of the traditional Chinese herbal medicine TSF have been described in other reports [17, 21], including the original study [22] on which this post-hoc analysis and cross-sectional study were based. Results from the original study indicated that TSF as an adjunct to conventional diabetes treatments was more efficacious in lowering 24 h UP than conventional treatments alone in patients with macroalbuminuria [22]. Evidence from our previous studies have revealed that TSF attenuates development of diabetic nephropathy [17, 18, 22]. Specifically, TSF improves renal impairment by decreasing accumulation of extracellular matrix in the renal tissue by reducing expression of transforming growth factor-beta1 (TGF-β1) and increasing expression of matrix metallopeptidase 9 [19]. We have also demonstrated that TSF can reduce glomerulosclerotic and interstitial fibrotic indices and decrease serum TC and 24 h UP [17]. In this post-hoc analysis, we found that TSF decreased UP over the time course in macroalbuminuria patients, but did not differ from placebo. This phenomenon might be explained by the small sample size, since there were only 15 participants in each group.

The renal protective mechanisms of individual herbs in TSF have been discussed in numerous studies. Active components in astragalus, rehmannia, rhubarb, cornelian cherry, and winged burning bush twig have been found to reduce albuminuria, suppress activation of nuclear factor-kappaB, down-regulate TGF-β1 expression, and decrease extracellular matrix accumulation [26–30].

In this post-hoc analysis and cross-sectional study, we tested the hypothesis that TSF combined with conventional treatment would lower urinary and serum L-FABP both in micro- and macroalbuminuria patients. Our hypothesis originated from results of other clinical studies that showed urinary L-FABP was correlated with severity of tubulointerstitial injury, which suggested that urinary L-FABP is a predictor for the deterioration of renal function in DKD [10, 11]. L-FABP as an effective endogenous antioxidant is important for free fatty acid (FFA) metabolism [31, 32]. When experimental mice were exposed to albumin-bound FFAs, tubulointerstitial damage was more severe than mice exposed to albumin alone, as unbound albumin is reabsorbed in the proximal tubules [33]. Our previous study showed that TSF has a lipid-lowering effect, suggesting that TSF exerts a protective effect in the tubulointerstitium [17].

It is not clear whether urinary excretion of L-FABP is correlated with urinary protein. Results from a prospective observational follow-up study showed that although urinary L-FABP level was correlated with severity of albuminuria in all stages of DKD, this was not the case for a subgroup of patients with eGFR > 60 ml/min/1.73 m^2^ [11]. Therefore, in our study, the effect of TSF on urinary L-FABP might be independent of albuminuria.

To date, albuminuria and eGFR are widely used as markers of DKD progression. However, these tests are imprecise because long before the onset of microalbuminuria in diabetic patients, hemodynamic changes occur in the glomeruli [34]. For example, results from the Developing Education on Microalbuminuria for Awareness of Renal and Cardiovascular Risk in Diabetes (DEMAND) study showed that 20.5 % of diabetic patients with decreased kidney function had normal albuminuria [35]. The National Evaluation of the Frequency of Renal Impairment cO-existing with NIDDM (NEFRON) study found that more than half (55 %) of type 2 diabetes patients with an eGFR < 60 ml/min per 1.73 m^2^ had normoalbuminuria [36]. In addition, estimated GFR is commonly used instead of direct GFR measurement, which compromises accuracy [37]. Thus, current tests to assess kidney function and damage are inadequate and new biomarkers that can monitor kidney function, injury and repair, and predict DKD risk are needed. Indeed, L-FABP has been the focus of intense study as a new biomarker for early diagnosis and prediction of DKD risk [10, 11, 38]. Moreover, the Ministry of Health, Labour and Welfare in Japan has approved urinary L-FABP as a tubular biomarker for monitoring DKD [39].

In our study, urinary L-FABP levels increased with the progression of DKD, similar to other reports such as by Chou and colleagues [40]. However, we did not test urinary creatinine level, and as such were unable to use it to calibrate urinary L-FABP levels, which might be affected by the concentration of the urine samples. In addition to the kidney, L-FABP is expressed in the liver. L-FABP derived from the liver is released into the circulation, filtered through glomeruli and taken up by proximal tubules [41]. But, in our study no difference in plasma L-FABP levels were observed among participants in different stages of DKD, suggesting that plasma L-FABP levels do not affect urinary L-FABP levels.

Our study revealed that TSF could reduce the urinary L-FABP level in DKD patients, and has the same trend as urinary protein. Furthermore, since urinary L-FABP levels have been found to be significantly correlated with urinary 8-OHdG levels [12], our finding suggests that the beneficial effect of TSF in DKD is partly mediated by reduction of oxidative stress.

Our study has limitations. Healthy participants were not included. As such, differences in urinary L-FABP levels between DKD patients and healthy individuals were not evaluated. In addition, as a tubular marker, urinary L-FABP does not predict a decline in GFR in overt DKD patients [42]. Since the progression of DKD is defined as an increase in urinary albumin concentration, it may be difficult to determine the potency of urinary L-FABP for diagnosis or prediction of progression of DKD. Further research is needed to validate the reference values of urinary L-FABP for distinguishing different stages of DKD, as well as the possible mechanism by which TSF affects urinary L-FABP.

## Conclusions

In summary, results of this post-hoc analysis and cross-sectional study further extend observations that the traditional Chinese medicine Tangshen Formula appears to be a promising treatment for DKD. Moreover, urinary L-FABP may be a useful biomarker for predicting the progression of DKD. Further studies are needed to confirm its potential for clinical application.

## Abbreviations

24 h UP, 24-h urinary protein; A1C, glycosylated hemoglobin; ADA, American Diabetes Association; ALT, alanine aminotransferase; AST, aspartate aminotransferase; BMI, body mass index; BUN, blood urea nitrogen; DEMAND, Developing Education on Microalbuminuria for Awareness of Renal and Cardiovascular Risk in Diabetes; DKD, diabetic kidney disease; DM, diabetes mellitus; eGFR, estimated glomerular filtration rate; ESRD, end-stage renal disease; FBG, fasting blood glucose; FFA, free fatty acid; HDL, high density lipoprotein; IQR, interquartile range; LDL, low density lipoprotein; L-FABP, liver-type fatty acid binding protein; NEFRON, National Evaluation of the Frequency of Renal Impairment cO-existing with NIDDM; NKF-KDOQI, National Kidney Foundation Kidney Disease Outcomes Quality Initiative; Scr, serum creatinine; TC, total cholesterol; TCM, Traditional Chinese Medicine; TG, triglyceride; TGF-β1, transforming growth factor-beta1; TSF, Tangshen Formula; UAER, urinary albumin excretion rate
